# Enhanced Surface Determination beyond Photoemission via Auger Photoelectron Coincidence Spectroscopy

**DOI:** 10.1021/acs.jpclett.4c01745

**Published:** 2024-08-02

**Authors:** Danilo Kühn, Swarnshikha Sinha, Fredrik O. L. Johansson, Ruslan Ovsyannikov, Andreas Lindblad, Alexander Föhlisch, Nils Mårtensson

**Affiliations:** † Institut für Methoden und Instrumentierung der Forschung mit Synchrotronstrahlung, Helmholtz-Zentrum Berlin für Materialien und Energie GmbH, Helmholtz-Zentrum Berlin GmbH, Albert-Einstein-Str. 15, 12489 Berlin, Germany; ‡ Uppsala-Berlin Joint Laboratory on Next Generation Photoelectron Spectroscopy, Albert-Einstein-Str. 15, 12489 Berlin, Germany; ¶ Department of Physics and Astronomy, Division of X-ray Photon Science, Uppsala University, P.O. Box 256, 751 20 Uppsala, Sweden; ⌀ Institut des NanoSciences de Paris, INSP, Sorbonne Université, CNRS, F-75005 Paris, France; ∥ Institut für Physik und Astronomie, Universität Potsdam, Karl-Liebknecht-Straße, 14476 Potsdam, Germany

## Abstract

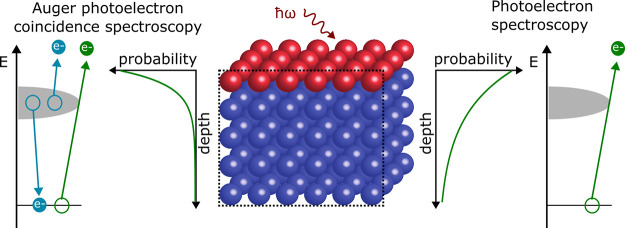

The study of surface properties at the nanoscale plays a crucial role in material science applications. This paper demonstrates the capabilities of Auger PhotoElectron Coincidence Spectroscopy (APECS) to obtain data with varying surface sensitivities from a single measurement. This makes it possible to extract the spectrum from the outermost surface layer even when faced with strongly overlapping surface and bulk spectral features, which we demonstrate by accurately extracting the surface component in Au 4f photoemission. Leveraging high energy resolution, transmission efficiency, tunable photon energy, and remarkable surface sensitivity of the APECS setup, we propose that optimal experimental conditions can be tailored to determine surface spectra accurately for a diverse range of materials. This opens new avenues for advancing our understanding of nanoscale surface phenomena across various material systems.

Many of the unique characteristics of nanoparticles and nanostructured systems are linked to their large surface to volume ratios. Core-level photoelectron spectroscopy (PES) is one of the most powerful and widely used technique to study the electronic structure and chemical properties of surfaces and interfaces.^1−4^ From the core-level energies, one can in a quantitative way determine what elements are present in the surface region of a sample. Furthermore, from the detailed core-level binding energies one can derive information about the chemical state of the probed atoms (chemical shifts).

The photoemission signal originates from the outermost atomic layers of the sample, with an intensity which is exponentially attenuated as a function of depth. In many cases it is necessary to derive more detailed information about the depth distribution of the various spectral contributions and in particular to determine the spectrum originating from the outermost atomic layer. This requires that one can perform measurements with different surface sensitivities, which is usually done by varying the emission angles of the photoelectrons or by changing their energies by tuning the photon energy. A common difficulty for these techniques is that a number of other parameters are changed at the same time. An alternative technique is provided by Auger PhotoElectron Coincidence Spectroscopy (APECS).^5−13^ In the present paper we demonstrate how one, from the same APECS data set, can derive photoelectron spectra with different surface sensitivities. The spectrum containing all photoemission events is compared to the subset of the data for which an Auger electron has been detected in coincidence. The surface sensitivity of the latter spectrum is further enhanced due to the attenuation also of the Auger signal.^7,9,14^ In this way two spectra can be compared where the surface sensitivity is the only difference, all other experimental parameters are identical. This capability to separate the surface and bulk spectral contributions is demonstrated using the 4f core level spectrum for gold for which there is a Surface Core Level Shift (SCLS) for the outermost atomic layer.^15−18^

One of the most important aspects of PES is its surface sensitivity. When excited with soft X-rays, only a few atomic layers contribute to the spectrum. The photoelectron intensity is exponentially attenuated due to inelastic losses in the sample.^19−22^ The fundamental parameter which defines the surface sensitivity is the Inelastic Mean Free Path (IMFP) for the emitted electrons. It has usually a minimum in the range of 50–100 eV and is longer both at lower and at higher kinetic energies. To a first approximation, it is often described in terms of the so-called Universal Mean Free path curve.^23^ However, for a more detailed analysis one has to take into account that the IMFP is dependent on the specific material. The IMFP has been calculated for a number of systems^24−26^ and in many cases there are also experimental determinations.^27−29^ If elastic scattering effects are small enough to be neglected,^20^ then the electron intensity of the nonscattered electrons *dI* from a certain depth *z* measured vertically from the surface is
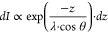
1Here λ is the IMFP and θ the emission angle measured with respect to the surface normal. For a particular material and for a specific sample geometry it is convenient to discuss the surface sensitivity in terms of the mean escape depth (MED): Δ = λ · cos θ, which is defined as ”the average depth normal to the surface from which the electrons escape”.^22^

In order to get more detailed information on the depth distribution of the different species in a sample, it is necessary to vary the mean escape depth (Δ) of the experiment.

One technique is to vary the angle of emission for the photoelectrons.^30^ To a first approximation, this leads to a cos θ variation of the mean escape depth. However, if the sample is not atomically flat, the angle of emission becomes a function of the local surface orientation, which leads to an unknown distribution of emission angles, making it difficult to estimate MED’s for a particular experimental geometry.^31^ If there is crystalline order, there are furthermore electron diffraction effects which will cause deviations from the simple cos θ dependence. Also the effects of elastic scattering are angular dependent,^20^ making a quantitative analysis based on the emission angle even more complex. Especially for a structurally complex sample, like for instance a sample consisting of nanoparticles, this technique is of limited use.

A different technique is to vary the energy of the exciting radiation.^30^ In this way the kinetic energies of the emitted electrons are varied, leading to a different mean escape depth. However, changing the photon energy has also other consequences. For large changes, the spectral resolution may vary. Any electron diffraction effects will also be affected. There are changes in the relative cross sections when the emission from different species are compared. Even for the same element in different local environments, the relative cross sections may be energy dependent, due to EXAFS-like variations.^32^

One way to drastically increase the surface sensitivity of a core electron photoelectron spectrum is to record the spectrum in coincidence with an Auger transition originating from the same core level.^7,9,14^ The coincidence spectrum is not only attenuated due to the inelastic scattering of the photoelectrons but also due to the attenuation of the Auger signal. In particular, when using a synchrotron radiation source one can chose a photon energy such that the kinetic energy of the photoelectrons is close to the minimum of the IMFP. If there is also an Auger transition from the same core level which has a kinetic energy close to the IMFP minimum, a very high surface sensitivity can be achieved. One can then compare two spectra, derived from the same APECS measurement, which have different surface sensitivities. In one case all photoelectrons are included in the spectrum while in the other case only those electrons which are coincident with an Auger electron are used. This implies that all experimental parameters are the same for the two spectra.

In order to quantify the surface sensitivity of APECS, the mean escape depth of a single electron probe (as defined above) can be extended to the case of coincident pairs of Auger- and photoelectrons Δ_pair_, i.e. ”the average depth normal to the surface from which the coincident electron pairs escape”. Since the depth dependent electron pair intensity *dI*_pair_ of nonscattered electron pairs is the product of the individual probabilities, we have
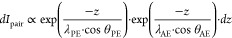
2

The MED of the pairs of Auger and photoelectrons is then:
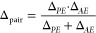
3

In the common case of similar individual MED’s, it is Δ_pair_ ≈ 0.5 · Δ. In the following we will use APECS to obtain quantitative MED’s and IMFP’s of gold.

The experiment was conducted at the CoESCA station for electron–electron coincidence spectroscopy at the UE52-PGM undulator beamline at the BESSY II synchrotron.^33^ The end station is equipped with two angular resolving time-of-flight spectrometers (type ARTOF by Scienta Omicron).^34,35^ These spectrometers provide excellent energy resolution and high transmission due to the wide angular acceptance of up to 60° (full cone). This enables, in combination with the pulse-picking by resonant excitation technique (PPRE)^36^ developed for time-of-flight applications at BESSY II (see SI for details), very efficient electron pair spectroscopy. Both spectrometers are oriented in a horizontal plane together with the photon beam. The sample normal is in the same plane with an angle of 10° to the photon beam, making it an almost normal incident geometry. The angle of the spectrometer recording the PE to the sample normal is 44° and the angle of the spectrometer recording the AE to the sample normal is 64°, see Figure 1.

**Figure 1:**
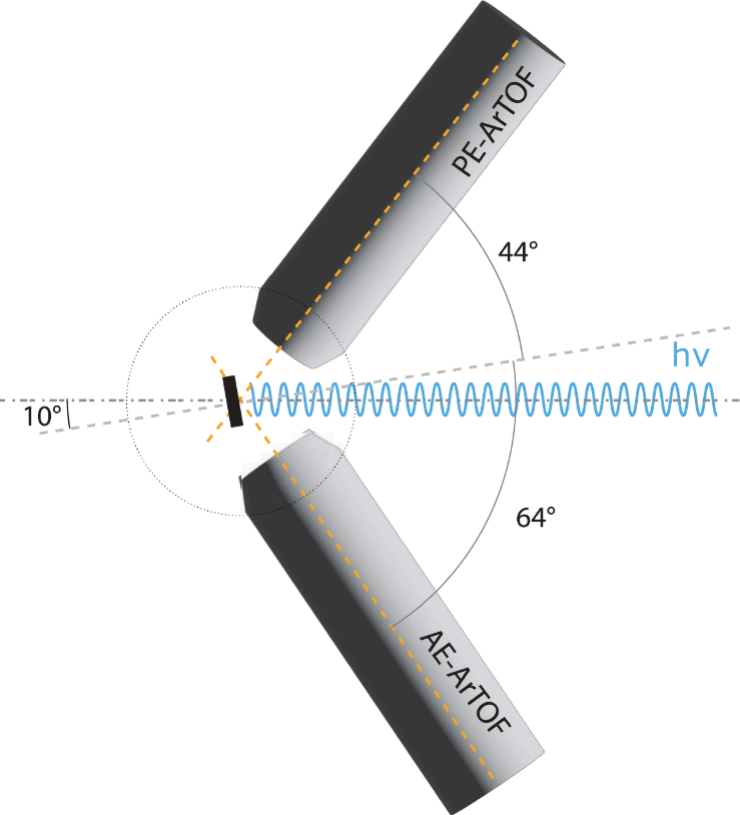
Experimental geometry of the APECS setup (see text for details).

We used a polycrystalline gold foil, which was cleaned in vacuum by repeated cycles of ion sputtering and annealing (see SI for details). Figure 2 shows an XPS scan from 30 to 220 eV kinetic energy recorded at *E*_ph_ = 220 eV. Besides the prominent Au 4f lines at 130 eV kinetic energy, the Au NVV Auger decay is clearly visible around 67 eV kinetic energy. For APECS, the spectrometers are operated in fixed-mode at a fixed center energy (*E*_cen_), which defines the energy window that can be analyzed simultaneously. The first spectrometer measures the photoelectrons and is operated in a lens mode with ±26° angular acceptance and an energy window size *E*_range_ of 4% of the center energy, giving a window of about *E*_range_ = 5.2 eV at *E*_cen_ = 131.6 eV. The second spectrometer measures the Auger electrons and is operated in a different lens mode with ±24.5° angular acceptance and an energy window size of 7% of the center energy, giving a window of about *E*_range_ = 4.7 eV at *E*_cen_ = 67 eV. The energy ranges measured simultaneously in these settings are depicted in Figure 2. The energy resolution of the AE and PE spectra is estimated to be 0.1 ± 0.02 eV in both cases (see SI for experimental details).

**Figure 2:**
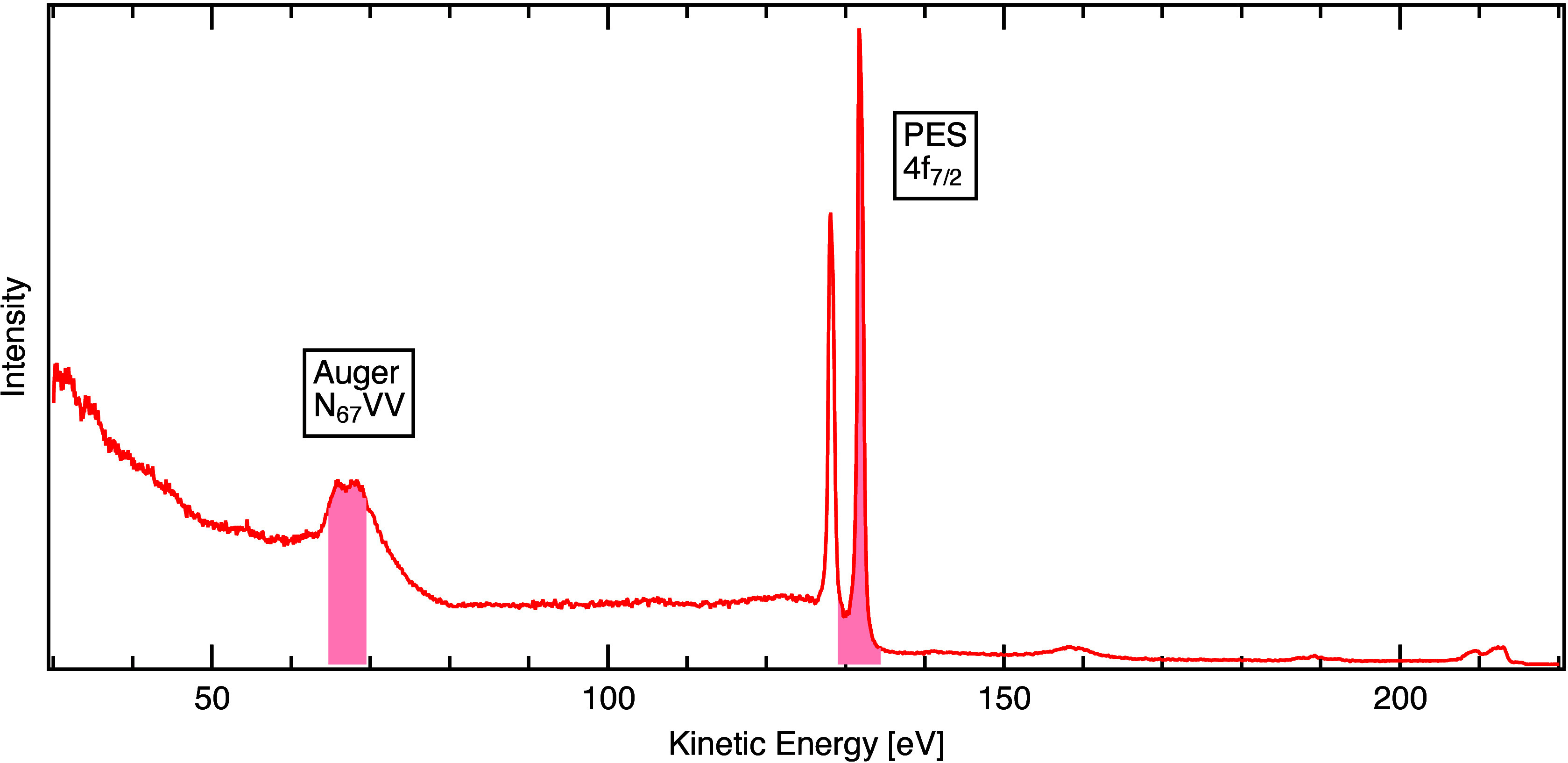
Photoemission spectrum of gold recorded at 220 eV photon energy. Shaded red regions mark the AES and PES energy windows of the APECS measurement. At higher kinetic energies one can observe photoemission from 5p, 5s and the valence band.

Figure 3a shows the noncoincident (conventional) Au 4f_7/2_ photoemission spectrum and the same spectrum in coincidence with N_7_VV Auger electrons. Since the spectrometers detect and save individual Auger- and photoelectron events with full temporal information, the coincident and noncoincident spectra can be obtained from the same measurement.^33^ The total acquisition time was about 10 h, leading to 4 · 10^6^ total counts in the noncoincident and 6.5 · 10^5^ total counts in the coincident spectra. The count rate of true coincidences is 18.6 counts per second at an accidental-to-true ratio of 3.4.^33^ Besides the Au 4f_7/2_ bulk component at 131.2 eV kinetic energy (84.0 eV binding energy), a clearly resolved surface component is seen, shifted by about 0.4 eV to lower binding energy.^15−18,37^ The two spectra in Figure 3a are identical in all respects, except that the surface to bulk intensity ratio is larger for the spectrum measured in coincidence with the Auger electrons. Also a very small nearly constant background arising from inelastically scattered electrons from shallow energy levels appears in the noncoincident spectrum. This implies that one can do a global least-squares fit (Igor Pro software^38^) of the two spectra with very few free parameters.

**Figure 3:**
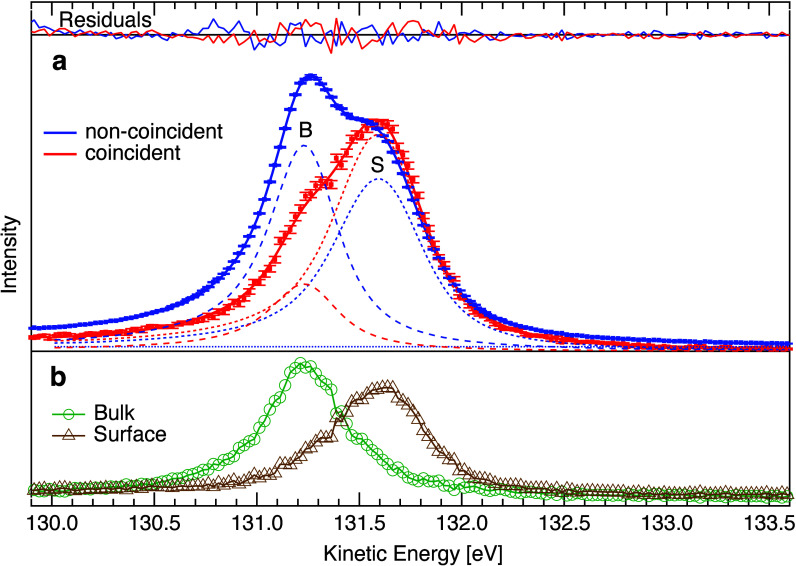
Au 4f_7/2_ from APECS: (a) Dotted curves with error bars show Au 4f_7/2_ photoemission data; (blue) noncoincident, (red) in coincidence with N_7_VV Auger electrons. The solid lines are fits to the data, and the dashed lines are the surface (S) and bulk (B) peak components. The small constant background in noncoincident data is shown. At top are residuals of the fits (scaled for better visualization). (b) Decomposition of APECS into surface and bulk components.

The spectra were fitted with two Doniach-Šunjić (DS) type peaks^39^ for the bulk and surface components and additionally a constant background for the noncoincident spectrum. All parameters, except for the intensities, are linked between the noncoincident and coincident spectra. Furthermore, the natural line width and DS asymmetry parameter are linked between the surface and bulk components. With this model we get the following results: Fractional surface signal (noncoincident) *f*_*nc*_ =  = 0.50 ± 0.02, fractional surface signal (coincident) *f_c_* =  = 0.79 ± 0.02, Δ*E*_*SB*_ = 0.37 ± 0.01 eV, natural line width *w*_*L*_ = 0.34 ± 0.01 eV (fwhm), asymmetry α = 0.06 ± 0.01, Gaussian width bulk *w*_*G*,*B*_ = 0.14 ± 0.01 eV (fwhm), Gaussian width surface *w*_*G*,*S*_ = 0.28 ± 0.01 eV (fwhm).

We find that two DS peaks give an excellent description of the data, as evident from the small residuals. The natural line width, the SCLS (Δ*E*_*SB*_) and peak asymmetry are in good agreement with previous investigations.^15−18^ The Gaussian width of the bulk peak is only slightly larger than the estimated experimental resolution. Hence, we see no sign of a second layer chemical shift. In strong contrast, the Gaussian width of the surface peak is significantly larger. We interpret this as due to surface sites with different coordination and consequently different SCLS for our polycrystalline sample.^16^

We can now use the obtained surface to bulk intensity ratios intensities to derive the MED’s. For this analysis we need to know that the Auger detection window does not selectively favor bulk or surface events. In certain systems there are significant differences between the surface and bulk Auger spectra. It may then be possible to use the coincidence spectra to separate the surface and bulk photoemission features. For gold, however, any shift or any difference in shape between the surface and bulk Auger spectra are very small, see the Supporting Information. This assumption was further supported by the fact that we found no differences between the Auger spectra in the chosen window, when measured in coincidence with the surface or the bulk 4f_7/2_ photoelectron peak.

If we define the surface layer to have a rigid thickness *d*_*s*_, then integrating eq 1 over the surface layer gives *I*_*S*_ =  and with the fractional surface intensity *f* =  we have
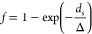
4

Using this formula and a surface layer thickness of *d*_*s*_ = 2.2 Å, calculated as an average layer spacing of the most stable gold surfaces (100) and (111),^15^ we obtain probing depths of Δ_*c*_ = 1.4 ± 0.1 Å and Δ_*nc*,*PE*_ = 3.2 ± 0.2 Å for the coincident and the noncoincident PES measurements, respectively. Hence, the MED of the coincident measurement is indeed less than a monolayer. The MED of the Auger electrons can in our case not be directly derived from surface fractional intensities. However, with Δ_*c*_ and Δ_*nc*,*PE*_ using eq 3 we can calculate it to be Δ_*nc*,*AE*_ = 2.5 ± 0.3 Å.

We will now compare our experimentally derived MEDs with theoretically calculated IMFPs from Shinotsuka et al.^26^ With Δ = λ · cos θ and the average emission angle of the photoemission signal θ = 44° we can estimate the IMFP of gold at *E*_kin_ = 130 eV from the MED of the noncoincident measurement and find λ = 4.4 ± 0.3 Å.^26^ This is in very good agreement with the theoretical IMFP of 4.7 Å. Accordingly, we obtain with the average emission angle of the Auger electron signal θ = 64° the IMFP of gold at *E*_kin_ = 70 eV and find λ = 5.7 ± 0.8 Å, again in fair agreement with the theoretical value of 4.8 Å.^26^

In the example above there were clearly shifted bulk and surface peaks and the separation of surface and bulk spectra becomes trivial. However, it is clear that the APECS technique can be used to isolate the surface spectrum even if this is not the case. First of all, a high surface sensitivity is reached for the coincidence spectrum, in many cases such that the surface signal even dominates. The bulk contribution can then be further reduced by subtracting a fraction of the more bulk sensitive, noncoincident, spectrum obtained from the same data set. Using estimated MEDs for the photoelectron and Auger electron spectra, respectively, one can rather accurately calculate what fraction of the noncoincident spectrum should be subtracted in order to remove the bulk signal. This procedure is demonstrated in Figure 3b with our gold spectra as an example. First, the coincident (*S*_*c*_) and noncoincident (*S*_*nc*_) photoemission spectra are normalized to have same area intensities. With the MEDs estimated from the independently known IMFPs and the surface layer thickness, one can with eq 4 calculate the surface fractional intensities of the coincident (*f*_*c*_) and noncoincident (*f*_*nc*_) PES. The pure surface (*S*_*S*_) spectrum is then calculated with eq 5, i.e. *S*_*nc*_ is subtracted from *S*_*c*_ after scaling the spectra with the respective surface fractional intensities. The bulk spectrum can be obtained accordingly.
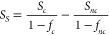
5

It is clear from Figure 3b that the surface and bulk spectra can be accurately decomposed using this procedure. There are of course uncertainties connected to the estimated intensity ratios. However, since the bulk contribution is relatively small already from the beginning in the coincidence spectrum, only a small intensity is subtracted. The uncertainties in the subtraction therefore lead to limited errors when related to the resulting surface spectrum. It should also be noted that in order to perform an accurate decomposition, all contributions other than the elastic photoemission lines of the element under investigation should be removed from the spectra before the decomposition. In particular the background in the noncoincident spectrum arising from inelastic losses of PE features at lower binding energy, secondary electrons and, if existent, photoemission features from other elements captured in the measured energy window should be removed.

In summary, we have shown how APECS provides a unique way to obtain subsets of data with different surface sensitivities from a single measurement, which enables to isolate and study the surface spectral contribution. Intensity ratios of the well-known surface and bulk Au 4f photoemission components have been determined by applying a global fit to the more bulk sensitive noncoincident and the more surface sensitive coincident data subsets, respectively, for the latter we obtain a fractional surface intensity of 0.79. This exceptionally high surface contribution implies a coincident mean escape depth of 1.4 Å, which is even smaller than the thickness of a single atomic layer. We also used the gold data to show how an APECS measurement can be used to separate surface and bulk contributions by making difference spectra of the subsets of the data, where the intensity scaling factors can be estimated from independently determined mean free path values. With our setup providing high energy resolution, high transmission, tunable photon energy and very high surface sensitivity we propose that experimental conditions can be found, in order to determine surface spectra accurately, for a wide range of materials, even in the case of strongly overlapping surface and bulk spectral features.

## Supporting Information Available

Additional details of the experimental setup, sample preparation, the Au N_7_VV Auger processes (PDF)
