# Supplementary material for: Enhanced Surface Determination beyond Photoemission via Auger Photoelectron Coincidence Spectroscopy
Source: J Phys Chem Lett. 2024 Aug 2;15(32):8161–6. doi: 10.1021/acs.jpclett.4c01745 (PMC11331527; doi:10.1021/acs.jpclett.4c01745)
Supplement: Supplementary file 1 — jz4c01745_si_001.pdf [file jz4c01745_si_001.pdf]

Supporting information: Enhanced Surface Determination beyond Photoemission via Auger Photoelectron Coincidence Spectroscopy

Danilo Kühn[a],[*,†,‡] Swarnshikha Sinha[a],[†,‡] Fredrik O. L. Johansson,[¶,‡,§] Ruslan Ovsyannikov,[†,‡] Andreas Lindblad,[¶,‡] Alexander Föhlisch[b],[*,†,‡,‖] and Nils Mårtensson[b*,¶,‡]

†*Institut für Methoden und Instrumentierung der Forschung mit Synchrotronstrahlung, Helmholtz-Zentrum Berlin für Materialien und Energie GmbH, Helmholtz-Zentrum Berlin GmbH, Albert-Einstein-Str. 15, 12489 Berlin, Germany*

‡*Uppsala-Berlin Joint Laboratory on Next Generation Photoelectron Spectroscopy, Albert-Einstein-Str. 15, 12489, Berlin, Germany*

¶*Department of Physics and Astronomy, Division of X-ray Photon Science, Uppsala University, P. O. Box 256, SE-751 05, Uppsala, Sweden*

§*Institut des NanoSciences de Paris, INSP, Sorbonne Université, CNRS, F-75005, Paris, France*

‖*Institut für Physik und Astronomie, Universität Potsdam, Karl-Liebknecht-Stra§e 24/25, 14476 Potsdam, Germany*

E-mail: danilo.kuehn@helmholtz-berlin.de; alexander.foehlisch@helmholtz-berlin.de; nils.martensson@physics.uu.se

[a]These authors contributed equally to this work
[b]These authors contributed equally to this work

Experiment and sample preparation

All measurements were conducted at the CoESCA station for electron-electron coincidence spectroscopy at the UE52-PGM undulator beamline at the BESSY II synchrotron.[1] Recently the setup was upgraded by replacing the first generation ARTOF 1 spectrometer with a second wide angle spectrometer (ARTOF 2 EW), which has improved the performance significantly, mostly due to an increase of solid angle acceptance by a factor of four. The time-of-flight spectrometers require a pulsed photon source with a temporal bunch length ¡ 100 ps (FWHM), for optimum performance, and a minimum pulse-pulse distance (depending on kinetic energy) of about 100-200 ns, which can be obtained at undulator beamlines of BESSY II by using the pulse-picking by resonant excitation technique (PPRE)[2] in standard multi bunch operation mode. For this experiment, two PPRE bunches were provided in the filling pattern (effective photon pulse repetition rate of approx. 2.5 MHz), which has further enhanced the measurement performance. The photon flux of the monochromatized light was about 10^9 ph/s. All measurements are recorded at room temperature with 220 eV photon energy at 35 meV bandwidth (FWHM) and linear horizontal light polarization.

The polycrystalline gold foil (99.99% purity, by Sigma-Aldrich) was first chemically cleaned with aceton and propanol in an ultrasonic bath. Then it was cleaned in UHV by cycles of Argon ion sputtering (1.5 keV, 5 mA emission current) and subsequent annealing to 600 °C for 3 minutes. The cycles were repeated until no other elements than gold are found in XPS.

Discussion of the N_7VV Auger spectra arising from the gold surface and bulk

We find no differences between the Auger spectra in the energy region used for the coincidence measurements when measured in coincidence with the bulk or the surface photoemission peaks. It is also expected that the differences between the bulk and surface Auger spectra are very small. The Auger spectra for the noble metals are known to be complex due to the localization tendencies for the valence d electrons[3] The Auger spectra will therefore contain spectral features characteristic of both localized and itinerant d8 double hole final states. For the localized final state in gold, the double hole energy has been calculated to be shifted by 0.39 eV to lower binding energies at the surface relative to the bulk.[4] Since also the initial core hole energy is shifted to lower binding energies at the surface, with an experimental value of -0.37 eV, the surface Auger shift for the localized $5d^8$ state is therefore estimated to be close to zero. For copper and silver the localized final states are clearly dominating the Auger spectra. It is plausible that this is the case also for gold, although possibly to a somewhat lesser extent. However, this has not been established experimentally. For the itinerant double hole state there will be a shift due to a narrowing of the surface density of states (DOS). This narrowing has been estimated from XPS experiments where it was found that the center of gravity of the valence band is shifted by 0.5 eV to lower binding energies (Citrin – Present Ref. 12). This would imply a shift of 1.0 eV to lower energies for the center of gravity for the self convoluted DOS. This implies an Auger shift of 1.0-0.37=0.6 eV to higher kinetic energies. This is very small compared to the total width of the self convoluted DOS which is about 10 eV for the 5d-band. Since the localized and the itinerant Auger final states shift in the same direction, with a difference of only 0.6 eV we also expect the interaction between the localized and the itinerant final states to be very similar at the surface and in the bulk. Based on these arguments we expect very similar Auger spectra for the surface and for the bulk.

References

(1) Leitner, T.; Born, A.; Bidermane, I.; Ovsyannikov, R.; Johansson, F.; Sassa, Y.; Föhlisch, A.; Lindblad, A.; Schumann, F.; Svensson, S., et al. The CoESCA station at BESSY: Auger electron-Photoelectron coincidences from surfaces demonstrated for Ag MNN. *J. Electron Spectrosc. Relat. Phenom.* **2021**, *250*, 147075.

(2) Holldack, K.; Ovsyannikov, R.; Kuske, P.; Müller, R.; Schälicke, A.; Scheer, M.; Gorgoi, M.; Kühn, D.; Leitner, T.; Svensson, S.; N. Mårtensson,; Föhlisch, A. Single bunch X-ray pulses on demand from a multi-bunch synchrotron radiation source. *Nat. Commun.* **2014**, *5*, 4010.

(3) G. A. Sawatzky, In *Auger photoelectron coincidence spectroscopy*; Briant, C., Messmer, R., Eds.; Academic press, inc., 1988; Vol. 30; pp 167–243.

(4) Mårtensson, N.; Hedegård, P.; Johansson, B. Auger energy shifts for metallic elements. *Physica Scripta* **1984**, *29*, 154.
